# A Cost-Efficient Multiwavelength LED-Based System for Quantitative Photoacoustic Measurements

**DOI:** 10.3390/s21144888

**Published:** 2021-07-18

**Authors:** Michalis Orfanakis, George J. Tserevelakis, Giannis Zacharakis

**Affiliations:** 1 Foundation for Research and Technology Hellas, Institute of Electronic Structure and Laser, N. Plastira 100, GR-70013 Heraklion, Greece; orfanak@iesl.forth.gr (M.O.); tserevel@iesl.forth.gr (G.J.T.); 2 School of Medicine, University of Crete, GR-71003 Heraklion, Greece

**Keywords:** sensing, photoacoustics, LED, unmixing

## Abstract

The unique ability of photoacoustic (PA) sensing to provide optical absorption information of biomolecules deep inside turbid tissues with high sensitivity has recently enabled the development of various novel diagnostic systems for biomedical applications. In many cases, PA setups can be bulky, complex, and costly, as they typically require the integration of expensive Q-switched nanosecond lasers, and also presents limited wavelength availability. This article presents a compact, cost-efficient, multiwavelength PA sensing system for quantitative measurements, by utilizing two high-power LED sources emitting at central wavelengths of 444 and 628 nm, respectively, and a single-element ultrasonic transducer at 3.5 MHz for signal detection. We investigate the performance of LEDs in pulsed mode and explore the dependence of PA responses on absorber’s concentration and applied energy fluence using tissue-mimicking phantoms demonstrating both optical absorption and scattering properties. Finally, we apply the developed system on the spectral unmixing of two absorbers contained at various relative concentrations in the phantoms, to provide accurate estimations with absolute deviations ranging between 0.4 and 12.3%. An upgraded version of the PA system may provide valuable in-vivo multiparametric measurements of important biomarkers, such as hemoglobin oxygenation, melanin concentration, local lipid content, and glucose levels.

## 1. Introduction

Over the last decade, extensive research has been conducted to develop novel photoacoustic (PA) based diagnostic systems for biomedical applications. PA sensing relies on the formation of acoustic waves following the absorption of intensity-modulated (typically pulsed) optical radiation by a material. More specifically, a portion of the absorbed optical energy is converted into heat via non-radiative molecular relaxation mechanisms, inducing a rapid thermoelastic expansion of the medium and the subsequent generation of an initial pressure that propagates in space as acoustic waves [1]. These waves, typically found in the MHz frequency regime, can then be recorded using the same detection equipment (e.g., piezoelectric elements) as in traditional ultrasound imaging. The amplitude of PA waves is directly proportional to the absorption coefficient of the medium for the employed excitation wavelength; therefore, the technique provides optical absorption information with high sensitivity [2]. The ability of PA sensing to provide molecular absorption specificity [3], and the low attenuation of PA waves [4], provide unique advantages towards developing novel systems for biomedical imaging and sensing applications that can obtain valuable diagnostic information deeper in highly scattering tissues when compared to pure optical imaging approaches [5]. In this direction, special emphasis has been given to developing reconstruction-based PA tomography (PAT) [6], PA microscopy (PAM) [7], or even hybrid imaging systems integrating both PA and optical [8,9,10], or ultrasound [11] techniques into a single instrument. Moreover, several spectral unmixing techniques [12,13] have been utilized with multispectral PA imaging systems to provide spectroscopic information for endogenous and exogenous chromophores in tissues. Even though PA systems present a rich potential for diagnostic biomedical applications, they are usually developed in the form of sensitive, complex, and bulky laboratory setups utilizing light pulses emitted by high-cost Q-switched nanosecond lasers, with limited wavelength availability. To extend PA applicability in both preclinical and clinical directions, it is necessary to enhance multispectral capabilities, increase portability and lower the cost of PA systems. Within this framework, the focus has been given lately on the development of PA systems based on laser diodes (LD) [14,15,16,17,18,19,20,21], and light emitting diodes (LED) [22,23,24,25,26,27] with promising perspectives. Such alternative PA excitation sources are usually cheap (sold at the cost of several tens of euros), widely commercially available, and have a compact size, paving the way for the development of highly portable PA systems. Furthermore, they are available with a variety of wavelengths [22], ranging from near-ultraviolet (NUV) to visible and near-infrared (NIR) parts of the optical spectrum. In addition, they can generate short light pulses using low-cost drivers [28] that can be rather easily customized based on specific application requirements. Even though such low-cost excitation sources emit optical pulses of relatively low energy (typically in the order of hundreds of nJ to tens of μJ compared to several mJ of Q-switched lasers), the low PA signal to noise ratio (SNR) can be compensated with sufficient averaging, provided that the dynamic biological system under investigation does not demonstrate rapid changes during the averaging time interval. Usually, in pulsed mode, LEDs and LDs can be overdriven with electric currents much higher than their nominal continuous wave (CW) operation without any apparent damage [23]. Furthermore, these sources do not require any active cooling system, as duty cycles are typically kept well below 1%. In this manner, high pulse repetition rates (PRR) of several KHz can be achieved, thus providing sufficient signal averaging at reasonable times for a substantial SNR improvement [22,23,24]. Pulse energy provided by overdriven LEDs is comparable to LDs [24], however, there are still some basic differences. LEDs spectral emission is wider than LDs due to the different principles of operation, nevertheless, LEDs usually come at a relatively lower cost. Therefore, the selection between LDs or LEDs depends on the application’s specific features, by taking into consideration critical factors, such as the system’s geometrical, acoustic, or optical configuration, maximum cost of equipment, and optical wavelength availability. Current progress in LD and LED-based PA imaging and sensing has been summarized in several review papers [29,30,31]. However, research focusing on the spectral quantitative measurement capabilities of LD and LED-based PA systems is still relatively limited. Focusing on PA sensing using multiwavelength LED excitation, there are a few research efforts [32,33], based on a commercially available hybrid PA-ultrasound imaging system [34,35]. This imaging system utilizes two LED arrays integrated with an ultrasound transducer (UT) array for signal detection and can record co-registered ultrasound and multispectral PA images in-vivo. Despite the very interesting capabilities and high potential of such a LED-based imaging system, there is still the need for developing compact, highly portable, and ultra-low-cost PA sensing devices, optimized for quantitative estimations of intrinsic tissue chromophores and biomarkers, such as blood oxygenation, melanin concentration, local lipid content, and glucose levels.

In this context, we present here a compact, cost-efficient, multiwavelength PA sensing system for quantitative measurements, based on the utilization of two high-power LED sources and a single-element piezoelectric UT for signal detection. By employing simple, low-cost, and commercially available elements, the developed prototype has the potential to provide a spectral PA estimation of optical absorbers’ concentrations in tissue-mimicking phantoms with adequate sensitivity and accuracy. In this direction, we have initially investigated the performance of the PA sensing system in terms of optical spectral emission and pulse characterization. Subsequently, we have systematically determined the dependence of recorded PA amplitude on absorber concentration and applied energy fluence, and explored the provided spectral unmixing capabilities for quantitative absorber concentration estimation in the phantoms. Finally, we discuss future aspects and potential technical improvements that could lead to optimized low-cost, compact, portable, or wearable devices providing highly accurate spectral PA measurements in clinical or preclinical settings.

## 2. Materials and Methods

### 2.1. LED-Based PA System Configuration

**Figure 1:**
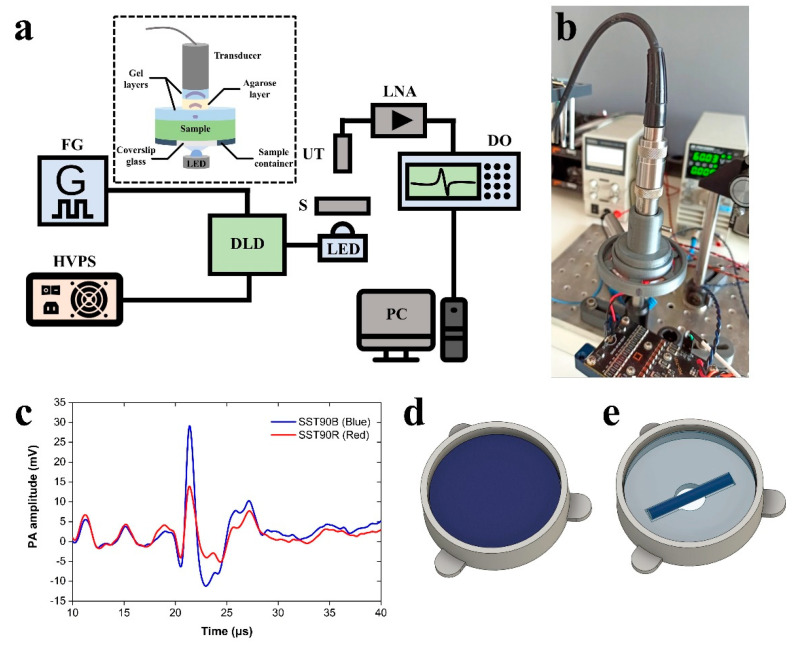
PA LED-based system for absorber unmixing. (**a**) Schematic representation of the developed PA apparatus. Inset shows a close-up of the basic configuration for quantitative PA measurements. Abbreviations: FG, function generator; HVPS, high-voltage power supply; DLD, diode laser driver; LED, light emitting diode; S, sample; UT, ultrasound transducer; LNA, low-noise amplifier; DO, digital oscilloscope; PC, recording computer. (**b**) Image of the LED-based system during the measurement of a phantom sample. (**c**) Typical PA waveforms were recorded from a gelatin phantom containing light green Indian ink (0.8% *v*/*v*), following the excitation with SST90B (Blue) and SST90R (Red) LEDs operating in pulsed mode. (**d**) 3D sketch of Series I phantoms characterized by strong optical absorption, but negligible scattering. (**e**) 3D sketch of Series II phantoms presenting appreciable optical absorption and scattering properties.

A schematic representation of the developed LED-based PA system is depicted in Figure 1a, whereas a respective image of the setup is shown in Figure 1b. Two high-power LED sources emitting in blue and red regions of the visible spectrum (SST90B (Blue) and SST90R (Red), Luminous Devices, Sunnyvale, CA, USA) are employed for the excitation of PA signals. The LEDs are overdriven using a commercial pulse diode laser driver (DLD) module (PCO7121, Directed Energy, San Rafael, CA, USA), which is further connected to a high-voltage power supply (HVPS) unit (9110 BK Precision, B&K Precision, Yorba Linda, CA, USA). A function generator (4050B BK Precision, B&K Precision, Yorba Linda, CA, USA) provides an external trigger signal to the DLD to control the pulse repetition rate, the optical pulse width, and synchronize signal recording (selected repetition rate: 1 KHz). Phantom samples are placed in a custom-designed 3D-printed container with a 10 mm diameter circular window at its bottom, which is sealed by a standard 22 × 22 mm^2^ microscope coverslip using a thin layer of waterproof silicone gel. A 3.5 MHz central frequency (−6 dB acoustic bandwidth: 2.44–4.61 MHz) unfocused single-element piezoelectric transducer (V383-SU, Olympus, Tokyo, Japan) is fixed at a standard distance of ~3 cm measured from the upper surface of the sample for detecting generated PA signals. A smooth and stable coupling between the transducer and the samples has been achieved by inserting a solid, ~2.5 cm thick, 1% *w*/*v* agarose layer (see inset in Figure 1a), improving substantially the repeatability of PA measurements. Thin layers of conventional ultrasound gel have been additionally applied on both sides of the agarose layer for better acoustic impedance matching. The intervening agarose layer serves as a delay line for detecting the PA signal, to be temporally discriminated from the unavoidable electronic noise that occurs when LEDs are triggered. Furthermore, it has provided uniform conditions for the transmission of the generated signals, thus enabling highly repeatable PA measurements. The detected PA waveforms (Figure 1c) are enhanced by an 80 dB low-noise RF amplifier (LNA-80dB-HF, Ciprian Sarl, Saint Ismier, Grenoble, France) and subsequently recorded by a digital oscilloscope (NI USB-5133, National Instruments, Austin, TX, USA; max. sampling rate 100 MS/s). Data are averaged 1024 times for SNR improvement before processing and storage using the NI free application software InstrumentStudioTM (National Instruments, Austin, TX, USA).

### 2.2. Phantom Sample Preparation

To investigate the capabilities of the developed LED-based PA system, two series of tissue-mimicking phantom samples were generated using gelatin as buffer medium, Indian inks as absorption agents, and intralipid to introduce optical scattering. The first phantom series (Series I) involved phantoms demonstrating predominant optical absorption properties with effectively negligible scattering. A gelatin solution of 5% *w*/*w* was initially prepared by mixing gelatin in powder form (Type A, Bloom 230) with deionized water. Two different types of Indian inks (Cobalt Blue 8 and Light Green 6, Pelikan, Hannover, Germany) were added at different amounts in the gelatin solution to form mixtures of various relative ink concentrations. The gelatin-ink mixtures were rigorously stirred for ~15 min to become homogeneous and were left aside for ~5 min to allow degassing of air bubbles that were formed during the process. Subsequently, 7 mL of the generated solutions were transferred to special 3D-printed cylindrical containers (Figure 1d) using a syringe and kept in the fridge at 6 °C for several hours to solidify. Containers were additionally covered with parafilm to prevent quick dehydration and degradation of the samples. The final diameter of Series I phantoms was 42 mm, whereas their height reached approximately 5 mm.

The second phantom series (Series II) was generated by preparing a solution of 5% *w*/*w* gelatin and 1% *w*/*w* intralipid (intralipid 20%, Fresenius Kabi, Bad Homburg, Germany) to introduce significant optical scattering properties. In this case, the 3D-printed container was designed to include a small channel of 2 mm in depth, 5 mm in width, and 30 mm in length. This channel was located ~1 mm above the upper surface of the glass coverslip, sealing the 10 mm diameter circular window. The intralipid-gelatin solution was initially injected carefully into the container, completely filling the volume between the channel and the coverslip glass. Solid ink-gelatin tubes (5 mm diameter; ~30 mm length) of various relative concentrations were carefully placed parallel to the channel, attaching to the intralipid-gelatin layer found beneath (Figure 1e). In this manner, the emitted LED radiation entering the phantom experienced optical scattering for a path of 1 mm prior to its strong absorption by the ink-gelatin tubes. The phantoms were placed in the fridge for several hours to solidify before filling the container with 7 mL intralipid-gelatin solution for the complete covering of the ink-containing tubes. Following the completion of this procedure, the phantoms were placed back in the fridge and left overnight. The developed system was sensitive enough to detect PA signals following excitation with both LEDs when ink concentration was as low as 0.5% *v*/*v* in Series II phantoms.

### 2.3. Spectral Unmixing Methods

A straightforward linear unmixing model was employed to quantify PA measurements in Series I phantoms demonstrating predominant optical absorption properties. By assuming that the Grüneisen parameter does not change significantly among different phantoms, it can be considered that the measured peak-to-peak PA amplitude *S*(*λ*) for a specific excitation wavelength λ is a linear combination of the molar extinction coefficients *ε_i_*(*λ*) of K distinct absorbers, weighted by their respective concentration *c_i_*

(1) $$S\left(\lambda \right)=A\Phi \left(\lambda \right)\overset{K}{\underset{i=1}{\sum }}\varepsilon_{i}\left(\lambda \right)c_{i}$$

Φ(*λ*) stands for the local energy fluence, whereas, A corresponds to a proportionality constant depending on the technical parameters of the experimental apparatus and the Grüneisen parameter of the samples. Alternatively, the molar extinction coefficient *ε_i_*(*λ*) can be expressed as a function of the measured optical absorption coefficient *μ_i_*(*λ*) for a known absorber’s concentration *C_ref_*, which is taken as reference, in the form,

(2) $$\varepsilon_{i}\left(\lambda \right)=\frac{\mu_{i}\left(\lambda \right)}{C_{ref}}$$

In this case, Equation (1) can be re-written as,

(3) $$S\left(\lambda \right)=A^{\prime }\Phi \left(\lambda \right)\overset{K}{\underset{i=1}{\sum }}\mu_{i}\left(\lambda \right)c_{i}$$

where *A’* is a new constant incorporating the reference concentration *C_ref_*. For the scope of this study, two types of absorbers were unmixed (Cobalt Blue 8 and Light Green 6, Pelikan Indian inks), requiring the use of two excitation wavelengths to determine the unknown concentrations *c*_1_ and *c*_2_. A weighted average optical absorption coefficient was estimated according to the measured ink spectra and the spectral emission of LED sources. In all cases, the average optical absorption values were found approximately equal to the respective absorption coefficients at the central wavelength of the LEDs. Therefore, absorption coefficients at the peak wavelength of LEDs emission were considered for subsequent analysis of PA measurements.

As regards Series II phantom samples demonstrating both optical absorption and scattering properties, the linear unmixing model incorporated a local energy fluence correction according to the effective optical attenuation coefficients of intralipid *μ_eff_*(*λ*). In this context, the local energy fluence Φ(*λ*) appearing in Equation (3) was estimated using an exponential decay model having the form,

(4) $$\Phi \left(\lambda \right)=\Phi_{0}\left(\lambda \right)e^{-\mu_{eff}\left(\lambda \right)z}$$

where Φ_0_(*λ*) stands for the measured fluence exactly before the intralipid-gelatin layer, while the path length z was set equal to 1 mm.

### 2.4. LED Performance Evaluation and Phantom Samples Optical Characterization

The current pulse provided to LED sources was measured through the integrated output of the DLD, whereas the emitted optical pulses were characterized using a Si-based photodiode (DET36A, Thorlabs, Newton, NJ, USA; rise/fall time: 14 ns). All data were recorded by the digital oscilloscope of the PA system (NI USB-5133). The emission spectra of LEDs were determined by a spectrograph (getSpec 2048, getAmo, Sofia, Bulgaria) using 1 nm wavelength steps. The average power of the LEDs during the pulsed operation was measured using a sensitive thermal power sensor head (S405C, Thorlabs, Newton, NJ, USA) connected to a USB power meter interface (PM100USB, Thorlabs, Newton, NJ, USA). For each measurement, the sensor head was placed at the bottom of an empty sample container, with its front side facing directly the 10 mm diameter circular window. In every case, the LED was positioned at a fixed distance below the surface of the coverslip glass. The two LED sources operated one at a time, and were placed at identical positions to achieve similar illumination conditions on the phantoms. The optical power on the sample was controlled using a set of neutral density filters (NDK01, Thorlabs, Newton, NJ, USA). Absorption spectra of phantom samples were measured by a UV-VIS spectrophotometer (BK-D590, Biobase, Jinan, China) using 1 cm path plastic cuvettes and a wavelength step of 1 nm.

## 3. Results

### 3.1. LED Operation Evaluation

**Figure 2:**
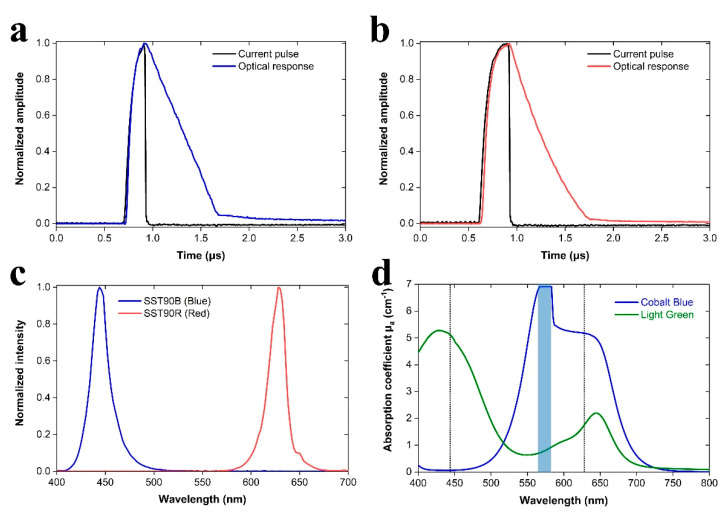
LED performance evaluation and phantoms optical characterization. (**a**) Current pulse provided to DLD and respective optical response by SST90B (Blue) LED (black and blue curves, respectively). (**b**) Similar data are shown for SST90R (Red) LED. (**c**) Emission spectra for the integrated LED sources. (**d**) Absorption spectra for ink-gelatin (Series I) phantom samples containing 0.8% *v*/*v* cobalt blue and light green Indian inks. Dotted lines indicate the central wavelengths of each LED’s emission. Blue-shaded stripe specifies the spectrophotometer’s saturation region (565-585 nm).

We had initially characterized the optical performance of LED sources when overdriven with the DLD and attempted to determine the optimum parameters for maximizing PA signal generation efficiency. After rigorous analysis of a wide range of values, we found out that an applied voltage of 60 V, corresponding to the maximum output of HVPS, provided current pulses of ~40 A to the LEDs without causing any noticeable damage after testing for long time intervals. Using this maximum voltage value, current pulses of gradually increasing durations were provided to the DLD at a constant repetition rate of 1 KHz, to maximize the peak power of the emitted optical pulse, thus achieving a good trade-off between adequate pulse energy and short optical pulse-width. It was observed that current pulses with an optimum Full Width at Half Maximum (FWHM) of 175 and 260 ns could provide approximately equal duration optical pulses of ~560 ns, when overdriving SST90B (Blue) (Figure 2a) and SST90R (Red) (Figure 2b) LEDs, respectively. Apart from the standard measurements of LEDs emission using a thermal sensor and a fast photodiode, these parameters were also directly tested on PA measurements using highly absorbing black tape samples, verifying the highest possible efficiency in signal excitation using the current configuration. Additionally, the spectra of LEDs emission were recorded using a spectrograph as explicitly presented in Figure 2c. The peak emission wavelength for SST90B (Blue) and SST90R (Red) LED was found at 444 and 628 nm, respectively, whereas the corresponding spectral width (FWHM) was ~20 nm for both sources. It must be mentioned that the measured peak emission wavelengths present a noticeable shift of several nanometers when compared to their respective nominal values under CW operation (455 and 621 nm, respectively), due to overdriving in pulsed operation.

### 3.2. Phantom Samples Optical Characterization

Prior to the realization of PA measurements, optical characterization of the generated phantom samples was performed using a UV-VIS spectrometer, evaluating the optical absorption features of the Indian inks at predefined concentrations. As gelatin presents a very small but not negligible optical absorption in the visible, a 5% *w*/*w* solid gelatin phantom was initially measured to obtain a reference absorption spectrum, which was subsequently subtracted from the measurements of ink containing phantoms, to provide the net optical absorption of Indian inks. Two 0.8% *v*/*v* ink-gelatin phantoms (Series I) containing cobalt blue and light green ink, respectively, were measured in the spectral region between 400 and 800 nm to estimate the optical absorption coefficient μ_a_ for each wavelength. The recorded absorption spectra (Figure 2d) reveal a broad absorption band between 530 and 670 nm for cobalt blue, accompanied by a strong peak in the region 565–585 nm, which caused the instrument to exceed saturation limits (blue-shaded stripe in Figure 2d). On the other hand, light green ink presented a different spectral pattern, characterized by high absorption in the region 400–490 nm, whereas a weaker absorption band appeared between 600–670 nm. The estimated absorption coefficients μ_a_ at the central wavelength of SST90B (Blue) LED (444 nm), were 0.06 and 5.1 cm^−1^ for cobalt blue and light green ink, respectively. As regards the central wavelength of SST90R (Red) LED (628 nm), the μ_a_ coefficients were found equal to 5.2 (cobalt blue) and 1.7 (light green) cm^−1^. Central wavelengths of LED sources are indicated with vertical dotted lines in the absorption spectra of Figure 2d.

### 3.3. Repeatability of PA Measurements

Aiming to evaluate the repeatability degree of phantom sample preparation procedure and respective PA measurements using the developed LED-based system, eight ink-gelatin phantoms were prepared (Series I) in total. Four of them contained cobalt blue ink at a concentration of 0.8% *v*/*v*, whereas the other four contained light green ink with similar concentrations. Subsequently, the peak-to-peak PA amplitude was measured for all phantom samples using both LEDs under identical experimental conditions. The coefficient of variation–CV% (standard deviation normalized to the mean value), as it is shown in Table 1., was ranging from 0.9 to 3.2% at maximum. This deviation among measurements is adequately low and indicates a high repeatability degree which constitutes a necessary precondition for the accurate spectral unmixing of absorbers using PA signals.

### 3.4. Parametric Study of PA Response

**Figure 3:**
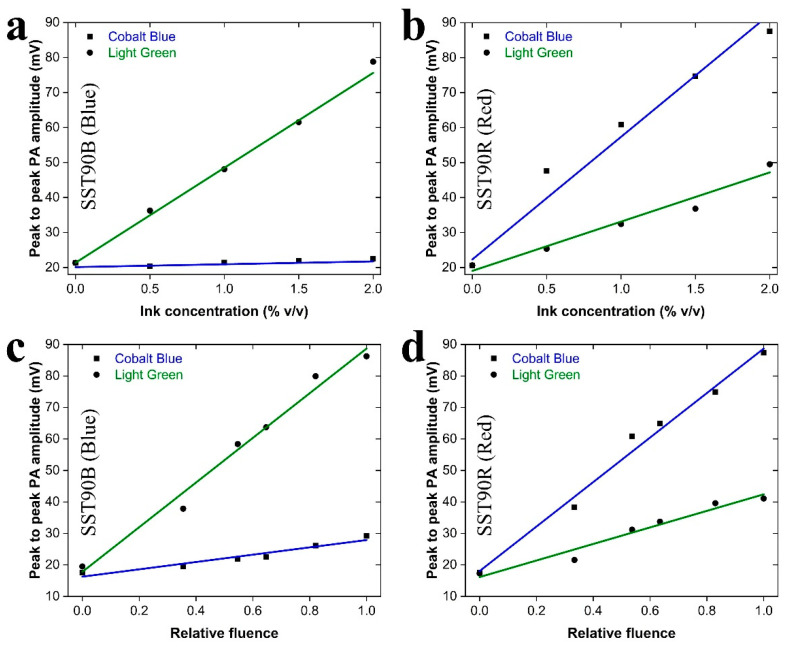
Parametric study of PA response versus absorber concentration and energy fluence. (**a**) A plot of measured peak-to-peak PA amplitude versus cobalt blue and light green ink concentration in Series I phantom samples using the SST90B (Blue) LED source. Blue and green lines correspond with linear fitting of the data. (**b**) Similar plot following PA excitation with the SST90R (Red) LED. (**c**) A plot of measured peak-to-peak PA amplitude as a function of applied relative energy fluence on 2% *v*/*v* cobalt blue and light green Series I phantoms using the SST90B (Blue) LED source. Blue and green lines correspond with linear fitting of the data. (**d**) Similar plot following PA excitation with the SST90R (Red) LED.

A parametric study of PA response as a function of (a) absorber (ink) concentration and (b) applied energy fluence, was conducted to evaluate the degree of linearity among the involved physical quantities, as predicted by the standard theoretical treatment of PA effect [1]. Such an investigation is crucial for the interpretation of PA measurements, as well as the extraction of reliable quantitative data using the developed LED-based PA system. In this context, we have generated five Series I ink-gelatin phantoms, containing Cobalt Blue Indian ink at gradually increasing concentrations between 0 and 2% *v*/*v* using a step of 0.5% *v*/*v*, whereas the same procedure was applied for another five phantoms containing light green ink. The peak-to-peak PA amplitude was subsequently measured for all phantom samples following excitation with SST90B (Blue) and SST90R (Red) LED sources, respectively, under identical experimental conditions (constant energy fluence). Figure 3a shows the recorded PA amplitude measurements as a function of ink concentrations using the SST90B (Blue) LED. Data have been fitted by two linear functions (green and blue lines), demonstrating an excellent linear relation between PA amplitude and light green concentration (R^2^ ≈ 1), but a concentration-independent behavior of PA signal generation regarding cobalt blue phantoms. This latter result is a direct consequence of the negligible absorption coefficient (0.06 cm^−1^) of cobalt blue at the central wavelength of SST90B (Blue) LED, indicating that the recorded low-amplitude PA signal is mainly generated, due to the slight optical absorption of gelatin. On the other hand, Figure 3b shows similar results using the SST90R (Red) LED, validating the highly linear behavior between PA response and ink concentration for both cases. The experimental procedure was identically repeated five times, providing highly consistent results with a maximum CV% value of 3.7% for all performed measurements. All estimated fitting parameters and goodness of fit (adjusted R^2^) values are explicitly presented in Table 2.

Having demonstrated the high linearity between the recorded PA amplitude and absorber concentration, we subsequently investigated the dependence of the PA signal on the applied energy fluence. Figure 3c shows the recorded PA amplitudes for 2% *v*/*v* cobalt blue and light green Series I phantoms using the SST90B (Blue) LED with a gradually increasing energy fluence on the sample. Linear fitting of the data has been performed (green and blue lines) to highlight the proportionality of the PA signal with the delivered relative fluence. Similar data are shown in Figure 3d for respective PA excitation using the SST90R (Red) LED source. The estimated fitting parameters and R^2^ values are presented in Table 3.

In this case, the experimental procedure was repeated five times, to obtain highly consistent results with a maximum CV% value of ~5%. The high R^2^ values of the linear fitting models, in combination with the apparent correspondence of the calculated slopes with the absorption coefficient values exhibited by the Indian inks as determined by independent measurements (see Figure 2d), highlight the strong linear behavior of PA amplitude in respect to the applied energy fluence on Series I phantoms.

### 3.5. PA Spectral Unmixing of Absorbers

**Figure 4:**
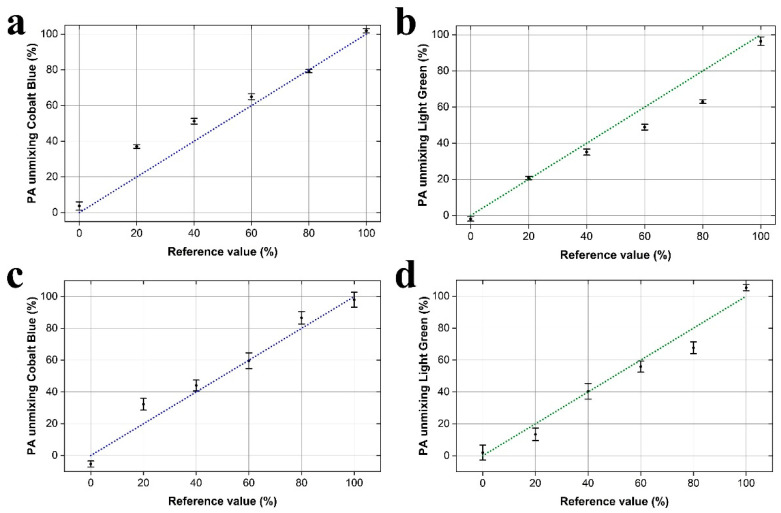
Quantitative measurements of relative absorber concentrations using the LED-based PA system. (**a**) Estimated concentrations of cobalt blue ink versus respective reference values in Series I phantoms. Error bars represent ±1 standard deviation out of four phantom measurements, while diagonal lines show the 1:1 correspondence. (**b**) A similar plot for light green ink concentrations. (**c**) Cobalt blue unmixing results for Series II phantoms, including optical attenuation effects from the intralipid layer. (**d**) Similar plot as regards the respective estimation of light green relative concentrations.

Having investigated the parameters and repeatability of PA signal excitation, the optical properties of the generated phantom samples, as well as the dependence of PA responses on absorber concentration and applied energy fluence, we finally explored the potential of the developed LED-based system on the accurate quantification of absorbers using tissue-mimicking phantoms. In this direction, we have generated two series of phantom samples exhibiting optical absorption only (Series I), or combined optical scattering and absorption (Series II) properties (see Section 2.2). Six different relative ink concentrations were selected for the realization of the unmixing experiments in each phantom series. The ink concentrations were ranging from 0.8% *v*/*v* cobalt blue and total absence of light green (100% cobalt blue-100B) to 0.8% *v*/*v* light green and total absence of cobalt blue (100% light green-100G), with four intermediate cases of ink mixtures at 20% intervals (i.e., 0.64% *v*/*v* cobalt blue and 0.16% *v*/*v* light green-80B20G etc.). Four phantoms were measured for each relative ink concentration ratio to provide quantitative PA spectral unmixing data using the reference absorption coefficients of the phantoms (see Section 3.2 in Results) and the direct linear unmixing model, as described in Section 2 (see Equation (3)). The required for the implementation of the linear unmixing model optical power ratio for SST90B (blue) and SST90R (red) LED sources were measured equal to 1.1. The power density of LEDs’ emission on the surface of the phantoms was measured at ~2.6 mW/cm^2^. Figure 4a shows the mean values of the obtained unmixing results versus different relative concentrations of cobalt blue ink in Series I phantoms (Reference values). Error bars represent ±1 standard deviation out of four phantom measurements, whereas the blue dotted diagonal line indicates a 1:1 correspondence of the unmixing results with respective reference values. To estimate the relative concentration of the ink, an offset has been subtracted from all peak-to-peak PA amplitude measurements, as determined using a pure 5% *w*/*w* gelatin phantom. In a similar fashion, Figure 4b demonstrates extracted results for light green ink relative concentration following the linear spectral unmixing procedures. The estimated relative ink concentration values for all measured Series I phantoms are explicitly presented in Table 4.

To evaluate the performance of the PA unmixing method in tissue-mimicking samples presenting both optical scattering and absorption properties, we investigated Series II phantoms, incorporating a 1 mm thick 1% *w*/*w* intralipid layer prior to the ink-gelatin (i.e., the absorbing) medium. Identical experimental parameters and procedures to Series I phantoms were also followed in this case, with the difference that a simple local fluence correction model (see Equation (4) in Section 2) was considered to compensate for the optical attenuation effects in the intralipid layer. The effective optical attenuation coefficients (*μ_eff_*) of 1% *w*/*w* intralipid were estimated at 0.296 and 0.255 mm^−1^ for 444 and 628 nm central wavelengths, respectively [36]. The results of the LED-based PA unmixing, regarding the cobalt blue ink relative concentration in Series II phantoms, are shown in Figure 4c. In a similar manner to Figure 4a,b, error bars indicate ±1 standard deviation out of four phantom measurements, whereas the blue dotted diagonal line reveals the 1:1 correspondence of the values. Respective data for light green ink are shown in Figure 4d, whereas the analytical spectral unmixing results for Series II phantoms are presented in Table 5.

## 4. Discussion and Conclusions

In this study, we have presented the spectral unmixing capabilities of a compact, cost-efficient LED-based PA sensing device for the accurate estimation of optical absorbers concentrations in tissue-mimicking phantoms. The developed prototype demonstrates the promising potential for developing highly portable PA systems that can be easily optimized according to the requirements of a specific application. The system is based on low-cost commercially available single-LED emitters, a single element PZT ultrasound transducer, and electronics that could be further integrated together for developing an upgraded, compact, low-cost LED-based PA sensor system able to provide reliable recordings of several biomarkers. Even though several studies have employed single-LED PA excitation for point measurements [22], reconstruction-based tomography [24], and microscopy [25] applications, this is the first time to our knowledge that spectral unmixing capabilities of a compact LED-based PA sensing device are systematically demonstrated.

The accuracy of PA measurements in Series II phantoms exhibiting both optical scattering and absorption properties, has been sufficient to determine the relative concentrations of the absorbing inks with absolute differences from the reference values ranging from 0.4 to 12.3% at maximum. Considering the scattering properties of the intralipid layer in Series II phantoms, as well as the standard background noise of the system, a maximum penetration depth of ~4–5 mm can be potentially achieved. Despite the low-cost of the integrated components, the precision of the acquired data is comparable to similar studies utilizing multispectral PA measurements [33]. Considering the apparent high repeatability in phantom generation procedures and PA amplitude measurements of single-absorber samples, we believe that the observed deviations from the reference concentration values are predominantly related to the adopted linear unmixing model, which in many cases fails to deliver accurate information [12,37].

Indian inks have been extensively used as absorbing agents in tissue-mimicking phantoms as they can effectively simulate the optical absorption properties of hemoglobin or other intrinsic tissue absorbers [38,39]. In particular, absorption coefficients of the measured phantom samples at the central wavelengths of the LED sources (~5 cm^−1^) were directly comparable or even smaller than the respective actual values for oxy and de-oxy hemoglobin [40]. In addition, gelatin has also been widely used as a buffer medium for phantoms generation, which can be imaged by employing either optical or PA modalities [39]. Gelatin solutions offer the possibility to tune easily soft tissue-like properties (e.g., optical and mechanical features) by adding variable concentrations of light absorbing and optical scattering agents, such as the Indian inks and intralipid [24,36], that have been used in the current study.

Following the successful demonstration of absorber unmixing capabilities provided by the multiwavelength LED-based PA system, several technical improvements could be introduced to upgrade further its performance. High-power LEDs of various types and wavelengths can be tested to increase the sensitivity of PA detection, leading to the accurate quantification of absorbers concentration in real tissues. Furthermore, the presented experimental prototype could be redesigned in a more compact and robust version to provide highly repeatable measurements in real time. New phantoms with improved temporal and mechanical stability characteristics can be generated, facilitating the optimization of the system prior to real tissue investigations, also enabling the evaluation of its long-term performance and stability. Modern pulse oximeters used for blood oxygenation measurements are mostly based on pure optical oximetric techniques [41] that have become the gold standard, but still, have limitations mainly due to light scattering. Quantitative PA techniques could compensate for this issue, as recordings are based on the detection of ultrasonic waves, which are dramatically more transmissive in tissues [42]. Moreover, melanin quantification is usually approached by standard spectroscopic methods [43], lipids by techniques, such as nuclear magnetic resonance or mass spectrometry [44], while recent research indicates progress towards non-invasive glucose monitoring [45]. PA sensing techniques could be successfully utilized as alternatives for monitoring such tissue biomarkers [46,47], and the prospect for employing LED illumination sources in low-cost, compact PA systems is highly motivating and could be put in research focus. A technically upgraded version of the PA apparatus is anticipated to offer valuable capabilities regarding the extraction of in-vivo multiparametric information involving the measurement of important biomarkers, such as hemoglobin oxygenation, melanin concentration, local lipid content, glucose levels, etc. We believe that the most crucial factor for recording reliable data in such highly-demanding applications is the incorporation of more accurate spectral unmixing models that will take into account the non-linear nature of light propagation in soft tissues. In this direction, different light propagation models [48,49], and various unmixing algorithms [50,51] must be systematically tested and evaluated to improve substantially the precision of the measurements at a level that will allow for reliable biomarker monitoring in a clinical or preclinical setting.

## Figures and Tables

**Table 1:** Repeatability of peak-to-peak PA amplitude measurements.

LED Type	Ink Type	Mean Signal (mV)	St. Deviation (mV)	CV%
SST90B (Blue)	Cobalt Blue	53.2	1.3	2.4
SST90B (Blue)	Light Green	174.8	3.1	1.8
SST90R (Red)	Cobalt Blue	181.1	1.6	0.9
SST90R (Red)	Light Green	84.8	2.7	3.2

**Table 2:** Peak-to-peak PA amplitude versus ink concentration.

LED Type	Ink Type	Slope	Intercept	Adj. R^2^ Value
SST90B (Blue)	Cobalt Blue	11.64	16.27	0.91
SST90B (Blue)	Light Green	70.92	17.78	0.98
SST90R (Red)	Cobalt Blue	70.66	18.04	0.98
SST90R (Red)	Light Green	28.27	16.14	0.95

**Table 3:** Peak-to-peak PA amplitude versus relative energy fluence.

LED Type	Ink Type	Slope	Intercept	Adj. R^2^ Value
SST90B (Blue)	Cobalt Blue	0.81	20.10	0.05
SST90B (Blue)	Light Green	27.13	21.35	~1
SST90R (Red)	Cobalt Blue	35.06	22.29	0.98
SST90R (Red)	Light Green	14.08	14.08	0.96

**Table 4:** Spectral unmixing results for Series I phantoms.

Sample	Cobalt Blue %	Light Green %	St. Deviation	Absolute Diff. %
100B	101.9	−1.9	1.2	1.9
80B20G	79.3	20.7	0.9	0.7
60B40G	64.9	35.1	1.7	4.9
40B60G	51.1	48.9	1.6	11.1
20B80G	37	63	1.0	17
100G	3.7	96.3	2.3	3.7

**Table 5:** Spectral unmixing results for Series II phantoms.

Sample	Cobalt Blue %	Light Green %	St. Deviation	Absolute Diff. %
100B	98.0	2.0	4.7	2.0
80B20G	86.6	13.4	3.9	6.6
60B40G	59.6	40.4	4.9	0.4
40B60G	44.1	55.9	3.5	4.1
20B80G	32.3	67.7	3.7	12.3
100G	−5.4	105.4	1.9	5.4
